# Evaluation of the *in vivo* antimalarial activity of ethanolic leaf and stembark extracts of *Anthocleista djalonensis*

**DOI:** 10.4103/0253-7613.59924

**Published:** 2009-12

**Authors:** Anita S. Bassey, Jude E. Okokon, Emmanuel I. Etim, Francis U. Umoh, Emmanuel Bassey

**Affiliations:** Department of Chemistry, Faculty of Science, University of Uyo, Uyo, Nigeria; 1 Department of Pharmacology and Toxicology, Faculty of Pharmacy, University of Uyo, Uyo, Nigeria; 2 Department of Pharmaceutical and Medicinal Chemistry, University of Uyo, Uyo, Nigeria; 3 Department of Pharmacognosy and Traditional Medicine, University of Uyo, Uyo, Nigeria

**Keywords:** Antimalarials, antiplasmodial, *Anthocleista djalonensis*, *P. berghei berghei*

## Abstract

**Objective:**

To evaluate the *in vivo* antimalarial activities of ethanolic leaf and stembark extracts of *Anthocleista djalonensis* used traditionally as malarial remedy in Southern Nigeria in mice infected with *Plasmodium berghei berghei*.

**Methods:**

The ethanolic extracts of the *A. djalonensis* leaf (1000 – 3000 mg/kg/day) and stembark (220 – 660 mg/kg/day) were screened for blood schizonticidal activity against chloroquine-sensitive *P. berghei* in mice. The schizonticidal effect during early and established infections was investigated.

**Results:**

The *A. djalonensis* leaf extract (1000 – 3000 mg/kg/day) exhibited a significant antiplasmodial activity both in the 4-day early infection test and in the established infection with a considerable mean survival time, which was incomparable to that of the standard drug, chloroquine (5 mg/kg/day). The stembark extract (220 – 660 mg/kg/day) also demonstrated a promising blood schizontocidal activity in early and established infections.

**Conclusion:**

These plant extracts possess considerable antiplasmodial activities, which justify their use in ethnomedicine and can be exploited in malaria therapy.

## Introduction

Malaria is still a major public health problem in Nigeria and other tropical countries where transmission of the disease is rarely controlled. Poor and inadequate drainage system has encouraged breeding of mosquitoes, causing infection to spread. Herbal preparations are patronised by most people in urban areas for the treatment of malaria infections in spite of the availability of conventional antimalarial drugs. *Anthocleista djalonensis* A.Chev (Loganiaceae) is a medium-sized tree of West tropical Africa, 30 – 45 feet-high, with blunt spines on the unbranched, pale grey trunk and widespreading crown.[1] The stem, rootbark and leaves of *A. djalonensis* are used to treat malaria, jaundice, diabetes and abscesses.[1,2] The seeds, barks and roots are used in Nigeria by the Igbos as antipyretic, laxative and remedy for various stomach disorders.[3] It is used to cure epilepsy in Ghana[2,4] and in southern Nigeria, the leaves and stembark are used traditonally as malarial remedy.[4] Burkill[5] reported that the plant is used as febrifuge, abortifacient and pain killer. Okorie[6] isolated phthalide and xanthones from *A. djalonensis*. Onocha *et al.*[7] isolated monoterpene diol, djalonenol and iridoid glucoside djalonenoside (also sweroside). They also isolated a dibenzo-alpha-pyrone-djalonensone from this plant. Some of these compounds and their semisynthetic derivatives were found to be cytotoxic against the brain tumor-transformed fibroblasts.[8] Reports of antibacterial and wound healing activity[3,9] as well as *in vitro* anthelmintic activity[10] have been published. We have investigated the antiplasmodial activity of the ethanolic extract of the leaves and stembark to ascertain their ethnobotanical uses.

## Materials and Methods

### Plant material

The leaves and stembark of *A. djalonensis* (A.Chev) (Loganiaceae) were collected in August, 2007, from the Nyan forest in the Uruan area of Akwa Ibom State and authenticated by Dr. Margaret Bassey, a taxonomist in the Department of Botany, University of Uyo, Uyo, Nigeria. A voucher specimen of the plant was deposited in the Faculty of Pharmacy Herbarium, University of Uyo, Uyo. The plant materials were shade-dried and then powdered.

### Preparation of extracts

The dried and powdered leaves and stembark of *A. djalonensis* (1 kg each) were separately and exhaustively macerated in 70% ethanol for 72 h. The liquid extracts obtained were concentrated in vacuo at 40°C. The corresponding yields were 3.16 and 3.25%, respectively.

### Phytochemical screening

Phytochemical screening of the extracts was carried out employing standard procedures.[11,12]

### Animals

Swiss albino mice (21-32 g) of both sexes were obtained from the University of Uyo animal house, Uyo, Nigeria. The animals were housed in standard cages and acclimatized for a period of 10 days. The mice were maintained on standard pelleted diet and water *ad libitum*. Approval for the study was obtained from the Animal Ethics Committee, University of Uyo.

### Acute toxicity

Acute toxicity study of the extracts was carried out to determine the LD50 of the extracts using mice by the intraperitoneal route using the method of Lorke.[13]

### Parasite innoculation

The chloroquine-sensitive *Plasmoduim berghei berghei* strain was obtained from the National Institute of Medical Research (NIMR), Lagos, Nigeria, and was maintained in mice. The inoculum consisted of 5 × 10^7^ *P. berghei berghei*-parasitized red blood cells per milliliters. This was done by determining both the percentage of parasitaemia and the red blood cell count of the donor mice using a hemocytometer and diluting the blood with isotonic saline in the proportion indicated by both determinations.

### Drug administration

The drugs and the extract used in this study were orally administered with the aid of a feeding cannula.

### Evaluation of suppressive activity on early infection

A method described by Knight and Peters[14] was used. The animals were divided into eight groups of five animals each and administered with leaf extract of 1000, 2000 and 3000 mg/kg/day and stembark extract (220, 440 and 660 mg/kg) and chloroquine (phosphate salt; Sigma, USA) (5 mg/kg) was used as positive control and the negative control group received distilled water. Each mouse was inoculated on the first day (Do), intraperitoneally, with infected blood containing 1 × 10^7^ *P. berghei berghei*-parasitized red blood cells. The mice were treated daily from day 0 (immediately after infection) to day 3. On the fifth day of the test (day 4), a thin blood smear was made from the tail blood sample of each mouse and stained with Giemsa and the percentage of parasitaemia was determined by counting the number of parasitized erythrocytes out of 200 erythrocytes in random fields of the microscope. The mean chemosuppression was calculated as 100 [(A–B)/A], where A is the mean percentage of parasitaemia in the negative control group and B is the mean percentage of parasitaemia in the test group.

### Evaluation of curative activity on established infection (Rane test)

A modified method similar to Ryley and Peters[15] was used. On the first day (day 0), standard inoculum of 1 × 10^7^ *P. berghei berghei*-infected erythrocytes was injected intraperitoneally into the mice. Seventy-two hours later, the mice were divided into eight groups (A H) of five animals each. Groups A, B and C were administered, respectively, with 1000, 2000 and 3000 mg/kg of *A. djalonensis* leaf extract. *A. djalonensis* stembark extract (220, 440 and 660 mg/kg) was administered to groups D, E and F, respectively. Chloroquine (phosphate salt; Sigma) (5mg/kg) was given to group G, which served as the positive control, while the negative control, group H, was given distilled water. The drug/extract was given once daily for 5 days. Thin smears stained with Giemsa stain were prepared from the tail blood of each mouse daily for 5 days to monitor the parasitaemia level. The mean survival time for each group was determined in each group over a period of 30 days (D0D29).

### Statistical analysis

Data are reported as mean ± standard error of the mean (SEM) and were analyzed statistically using one-way ANOVA followed by the Tukey Kramer multiple comparison test and values of *P <* 0.01 were considered significant.

## Results

### Phytochemical screening

The results of the phytochemical screening of the plant extracts are summarized in Table 1.

**Table 1:** The combined results of the phytochemical screening of the extracts

	*Source of extract A. djalonensis leaf extract*	*Stembark extract*
Alkaloids	+	−
Cardiac Glycosides	−	+
De-oxy-sugars	+	+
Flavonoids	+	+
Phlobatanins	+	−
Saponins	+	+
Tannins	+	+
Terpenes	+	+
Anthraquinones	−	−

### Acute toxicity

The mice were treated intraperitoneally with a single dose of 1, 2, 3, 4 and 5 g/kg of either *A. djalonensis* leaf or stembark extract after being starved for 24 h. *A. djalonensis* leaf extract (1–5 g/kg) produced physical signs of toxicity 1 h after administration, which include writhing, decreased motor activity, gasping and body/limb tone. All the animals treated with 5.0 g/kg of the extract survived and the LD50 was calculated to be 5.0 g/kg according to Lorke's rule.

The stembark extract of *A. djalonensis* (1, 2, 3, 4 and 5 g/kg) produced physical signs of toxicity 30 min to1 h after administration. The signs include writhing, gasping, palpitation, ptosis, decreased respiratory rate and death. All the mice treated with 2.5 g/kg and above doses of the extract died. The LD_50_ was calculated according to Lorke's rule to be 2.23 g/kg.

### Suppressive test

Evaluation of the suppressive activity of *A. djalonensis* leaf extract during early infection shows that the leaf extract produced a dose-dependent chemosuppressive effect at the various doses employed in this study (1000, 2000, 3000 mg/kg/day), with a chemosuppression of 17.05%, 26.82% and 41.46%, respectively [Table 2]. Similarly, the ethanolic stembark extract of *A. djalonensis* produced a dose-dependent chemosuppressive effect at the different doses employed (220, 440 and 660 mg/kg), with a chemosuppression of 22.61%, 27.39% and 33.32%, respectively. The effects of these extracts were significant (*P*<0.05) when compared with the control. The standard drug, chloroquine (5 mg/kg/day), caused 81.37% suppression [Table 2].

**Table 2:** Suppressive activities of leaf and stembark extracts of A. djalonensis during early *P. berghei berghei* infection in mice (4- day test)

*Drug/extract*	*Dose*	*Average %*	*Average %*
			
	*(mg/kg/day)*	*parasitaemia*	*suppression*
A.djalonensis leaf extract	1000	22.67 ± 0.40*	17.05
	2000	20.00 ± 0.63*	26.82
	3000	16.00 ± 0.63*	41.46
Stembark extract	220	21.67 ± 0.08*	21.61
	440	20.32 ± 0.53*	27.39
	660	18.67 ± 0.70*	33.32
Chloroquine Distilled	5	4.67 ± 0.53*	81.37
water (control)	0.2ml	28.06 + 0.07	−

Data are expressed as mean ± SEM for five animals per group.

* *P* < 0.05 when compared with control

### Effect of extracts on established infection

**Figure 1:**
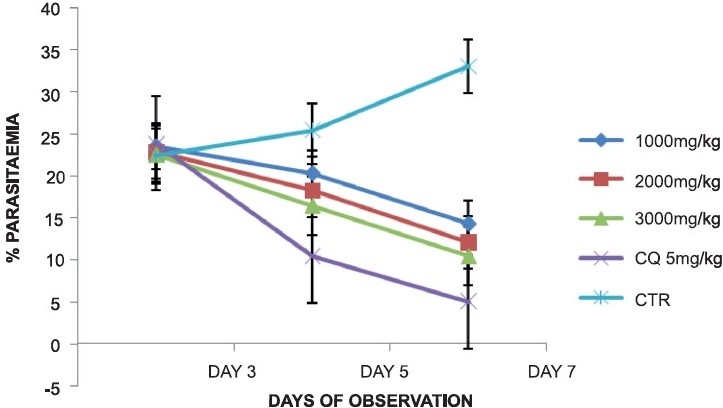
Antiplasmodial activity of the ethanolic leaf extract of *Anthocleista djalonensis* during early infection

**Figure 2:**
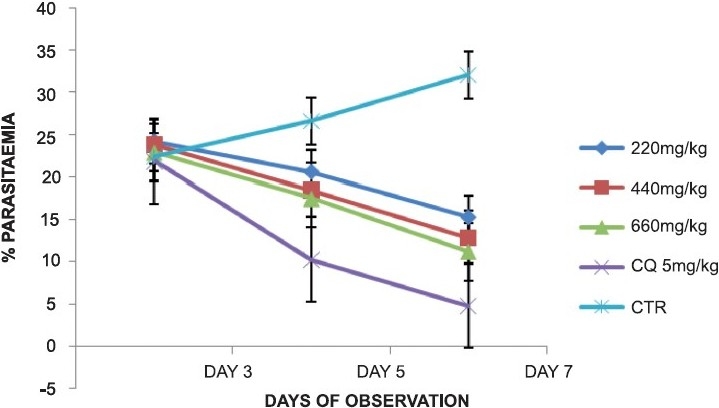
Antiplasmodial activity of the ethanolic stembark extract of *Anthocleista djalonensis* during early infection

Treatment of the *P. berghei*-infected mice with the plant extracts caused a dose-dependent daily reduction in parasitaemia in the extract-treated groups similar to that of the chloroquine-treated group, while the control group showed a daily increase in parasitaemia. In the groups treated with the leaf extract of *A. djalonensis* 1000, 2000 and 3000 mg/kg/day, the percentage of parasitaemia on day 7 was 14.3%, 12.0% and 10.4%, respectively, while the chloroquine-treated and control groups, respectively, had percentage parasitaemia of 5.0 and 33.0% [Figure 1]. The stem bark extract-treated groups had percentage parasitaemia of 15.3, 12.9 and 11.2%, respectively, for the 220, 440 and 660 mg/kg doses of extract-treated groups [Figure 2].

Data in Table 3 show that chloroquine (5 mg/kg/day) gave a mean survival time of 30.0 + 0.00 (mean + SEM) days compared to 17.67 + 1.70, 20.33 + 0.47 and 21.67 + 3.86 days, respectively, observed for the groups treated with 1000, 2000 and 3000 mg/kg of ethanolic leaf extract of *A. djalonensis*. Animals treated with 220, 440 and 660 mg/kg of *A. djalonensis* stembark extract had a mean survival time value of 16.33 + 2.08, 18.38 + 1.15 and 21.33 + 0.58 days, respectively. The mice in the control group survived for 14 days only.

**Table 3:** Mean survival time of mice receiving the various doses of ethanolic extracts of leaf and stembark extracts of *A. djalonensis* during an established *P. berghei* infection in mice

*Drug/extract*	*Dose (mg/kg/day)*	*Mean survival time (days)*
A.djalonensis Leaf extract	1000	17.67 ± 0.70*
	2000	20.33 ± 0.47*
	3000	21.67 ± 0.86*
Stembark extract	220	12.33 ± 0.15*
	440	16.33 ± 0.18*
	660	21.33 ± 0.58*
Chloroquine Distilled	5	30.0 ± 0.00*
water (control)	0.2 ml	13.67 ± 0.47

Data are expressed as mean ± SEM for five animals per group.

* *P*<0.05 when compared to control

## Discussion

The results show that *A. djalonensis* leaf and stembark extracts have moderate to negligible toxicity, as shown in their LD_50_ values of 5.0 g/kg and 2.23 g/kg for the leaf and stembark extract, respectively.[16] The results also show that the plant extracts possess significant antiplasmodial activity, as evident from the chemosuppressions obtained during the 4- day early-infection test. The leaf and stembark extracts also exhibited a significant curative effect during established infection less than to the standard drug, chloroquine (5 mg/kg/day), as demonstrated in the mean survival time of the mice in the extract- and chloroquine-treated groups. Although the antimalarial activities demonstrated by the leaf and stembark extract of *A. djalonensis* are low, these may have resulted from the crude nature of these extract and could be enhanced by further purification of these extracts. *A. djalonensis* leaf has been reported above to contain some phytochemical compounds like alkaloids, terpenes (monoterpenes) and flavonoids. Antiplasmodial screening of plants has implicated alkaloids, terpenes and flavonoids in this activity.[17,18] Sesquiterpenes and monoterpenes such as limonene have been implicated in endoperoxidation, leading to plasmodicidal activity.[19] The antiplasmodial activity observed with this plant could have resulted from its phytochemical constituents. Although the mechanism of action of these extracts has not been elucidated, some plants are known to exert antiplasmodial activity either by causing red blood cell oxidation[20] or by inhibiting protein synthesis,[21] depending on their phytochemical constituents. The extract could have exerted its action through either of the two mechanisms mentioned above or by some other unknown mechanism. These compounds may be acting singly or in synergy with one another to exert the antiplasmodial activity observed in this study. Thus, the active principle needs to be identified.

The results of the present study indicate that the extracts of the leaf and stembark extracts of the plant possess antimalarial activity. This confirms their use in ethnomedicine in the treatment of malaria. Therefore, it would be interesting if the active principles are isolated and characterized.
